# A Novel Nerve Block Technique for a Patient Undergoing Cardiac Device Implantation

**DOI:** 10.1016/j.jaccas.2022.08.028

**Published:** 2022-11-03

**Authors:** Pavel Antiperovitch, Ahmed T. Mokhtar, Muhtashim Mian, Raymond Yee, Habib Rehman Khan

**Affiliations:** a Department of Medicine, Division of Cardiology, London Health Sciences Centre, Western University, London, Ontario, Canada; b Department of Medicine, King Abdulaziz University, Jeddah, Saudi Arabia

**Keywords:** cardiac electronic device implantation, nerve block, pain control, AF, atrial fibrillation, CIED, cardiac implantable electronic device, PECS, pectoral nerve, SCN, supraclavicular nerve

## Abstract

A woman with type 1 myotonic dystrophy received an implantable cardioverter-defibrillator using a novel combination of ultrasound-guided supraclavicular nerve and pectoral nerve blocks. The entire procedure was completed without any procedural sedation or local anesthetic, and the patient did not experience any pain during or after the procedure. (**Level of Difficulty: Advanced.**)

## Central Illustration

**Figure:**
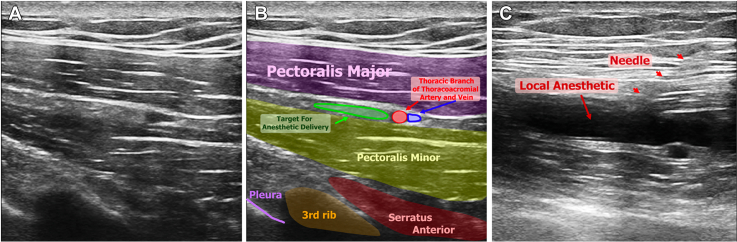

## History of Presentation

A 60-year-old woman with myotonic dystrophy type 1 and paroxysmal atrial fibrillation (AF) was evaluated for causes of presyncope. Her symptoms did not correlate to any time of day or activity and were sometimes associated with palpitations. Her vital signs were normal, and the rest of the physical examination was noncontributory.

**Learning Objectives**

• To describe the relevant neuroanatomy of the chest wall to a colleague or patient undergoing CIED implantation to ensure they understand and are comfortable with the procedure.
• To weigh the advantages and disadvantages of the nerve block procedure in patients undergoing CIED implantation to ensure appropriate patient selection.
• To perform the SCN and pectoral nerve block technique in patients undergoing CIED implantation to improve perioperative pain control.

## Past Medical History

The patient has a history of myotonic dystrophy type 1, paroxysmal AF, urinary incontinence, and postural hypotension.

## Differential Diagnosis

The patient’s presyncopal episodes could be due to bradycardia (atrioventricular block, slow AF, AF conversion pauses), tachycardia (ventricular tachycardia/ventricular fibrillation), and nonarrhythmic, such as postural hypotension.

## Investigations

Her 14-day Holter monitor did not capture any presyncopal spells but recorded AF 100% of the time. Her electrocardiogram demonstrated AF and a normal QRS morphology and duration. The echocardiogram revealed a mildly reduced left ventricular ejection fraction of 45% to 50%, and an enlarged left atrium. Laboratory investigations were unremarkable.

## Management

This patient had suspected AF conversion pauses as a cause of her symptoms, and a pacemaker was indicated. Based on the American Heart Association 2018 pacing guideline, patients with myotonic dystrophy type 1 with an indication for permanent pacing should receive a device with additional defibrillator capability.^1^ The patient had a significant risk of respiratory decompensation with intravenous sedation,^2^ and preferred insertion of an implantable cardioverter-defibrillator with local anesthetic only. We proposed a strategy of using a novel nerve block procedure and providing local anesthetic to areas outside nerve block coverage, to which the patient agreed. The institutional ethics review board approved this procedure as part of a larger study (Western & Lawson 2021-118630-56880).

**Figure 1:**
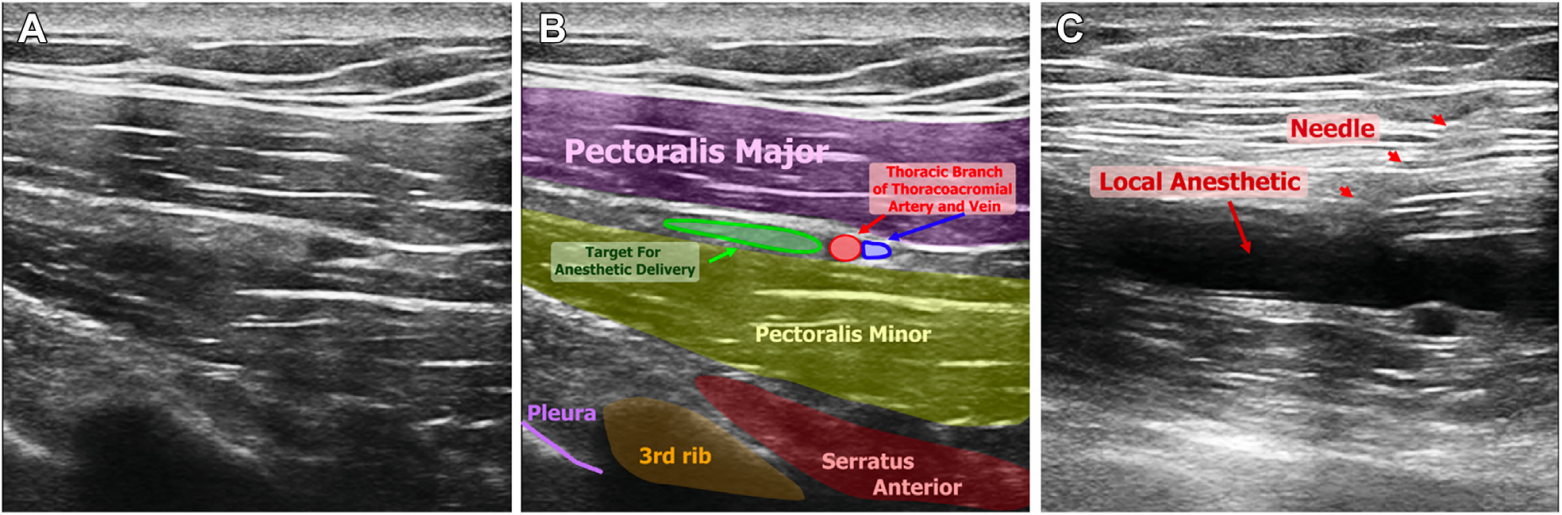
Ultrasound-Guided SCN Block **(A)** The ultrasound probe is placed on the lateral aspect of the neck in the sagittal plane posterior to the sternocleidomastoid muscle. The vertical landmark is the crossing of the external jugular vein with the posterior border of the sternocleidomastoid muscle at approximately one-third of the neck height. **(B)** Same image with a labeled overlay. **(C)** Approach to delivering local anesthetic in the area of the SCN taking care to keep the needle tip above the prevertebral fascia to avoid anesthetizing the brachial plexus. SCN = supraclavicular nerve.

The nerve block was performed at least 30 to 60 minutes pre-procedure to allow adequate time for the block to take effect. For the supraclavicular (SCN) nerve block, 20 mL of 0.5% bupivacaine, long-acting local anesthetic, was prepared (ropivacaine 0.5% is another option). A high-frequency linear ultrasound transducer (at least 13 MHz) was used to locate the SCN in the lower third of the lateral neck. The nerve is superficial to the prevertebral fascia above the middle scalene muscle (Figure 1). A 25-G needle was used to instill 3 to 5 mL of local anesthetic next to the nerve under direct ultrasound visualization.

**Figure 2:**
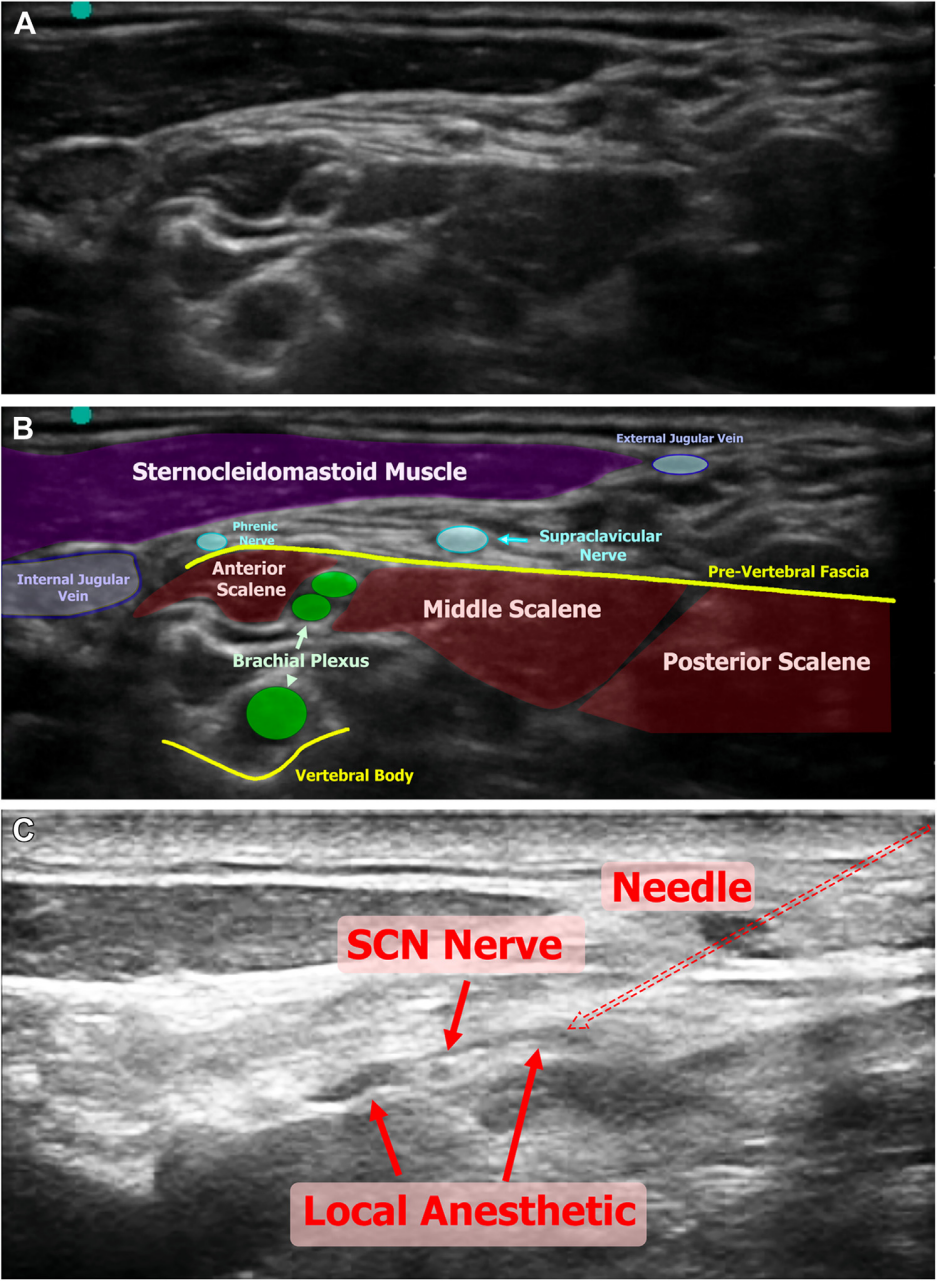
Ultrasound-Guided PECS 1 Block **(A)** The probe is positioned 2 inches below the clavicle in parallel orientation to the clavicle. The probe is then moved until the thoracic branch of the thoracoacromial artery is in view, which separates the pectoralis major and minor muscles. **(B)** Same anatomy with a labeled overlay. **(C)** Injection of the local anesthetic into the fascia between the pectoralis major and minor muscles taking care not to injure the blood vessels. PEC = pectoral nerve; SCN = supraclavicular nerve.

Pectoral nerve (PECS) 1 block was performed by placing the transducer 1 to 2 cm below the clavicle to visualize the thoracic branch of the thoracoacromial artery as a landmark lying in the space between the pectoralis major and minor muscles, as the nerves cannot be reliably visualized under ultrasound in this region (Figure 2). Approximately 15 mL of local anesthetic was injected into the plane between the pectoralis major and minor muscles, which distributes along the muscular plane to anesthetize the PECS. Small injections with fine needle adjustments were required to ensure anesthetic was injected into the interfacial layer, and not into the muscle.

After the nerve block procedure was completed, the patient was observed for 60 minutes, which allowed the anesthetic to take effect. Pre-procedural pin-prick test revealed dense and complete sensory loss over the infraclavicular zone, extending 5 cm below the clavicle, indicating a successful SCN block. Advancing the needle to the muscle confirmed a successful sensory block of the PECS 1. As both nerve blocks were successful, no additional local anesthetic was given up front.

The patient then underwent routine implantation of a dual-chamber implantable cardioverter-defibrillator in the left pre-pectoral area. The patient was comfortable and awake during the entire procedure and did not require local anesthetic or intravenous sedation. There were no complications after the procedure and the patient was discharged home 1 hour after implantation. When we contacted the patient post-procedure, she informed us that the skin overlying the incision was anesthetized for 8 hours after the nerve block, and there was no pain until the nerve block’s effect gradually diminished the following morning.

## Discussion

Currently, most cardiac implantable electronic devices (CIEDs) are inserted using intravenous midazolam and fentanyl for procedural sedation and subcutaneous infiltration of local anesthetic. However, some patients are poor candidates for intravenous sedation, such as those who have not fasted, had documented anesthetic intolerance/reactions, are hemodynamically unstable or elderly, and those with neuromuscular diseases.^3^^,^^4^ Nerve blocks have been previously reported to control postoperative pain in patients undergoing cardiac device implantation^5^, ^6^, ^7^; however, pain control during the implantation procedure has always required intravenous sedation and local anesthetic administration. Here, we introduce a novel combination of the SCN and PECS 1 blocks, which are easy to learn, safe to perform, and provide highly effective anesthetic for patients during and after CIED implantation.

The superficial structures of the skin and subcutaneous tissues are supplied by the SCN, a sensory nerve that originates from the C3/C4 nerve roots of the superficial cervical plexus. The deeper subcutaneous tissues and the tissue plane above the pectoralis major muscle are supplied by the medial and lateral PECSs, which can be anesthetized with a well-described PECS 1 block.^7^ The SCN block anesthetizes the skin to a depth of one-half of the subcutaneous tissue to allow a painless surgical incision, and the PECS 1 block allows deeper dissection and the creation of the CIED pocket.

This technique can be beneficial for patient flow by improving the experience of nonfasted patients who choose to undergo same-day procedure with local anesthetic alone. Patients presenting to the emergency department requiring a pacemaker can choose to undergo a same-day procedure with regional and local anesthesia. Outpatients who had not fasted may not need to be cancelled and rescheduled. Elderly patients can particularly benefit from the nerve block because intravenous sedation can result in acute delirium.^8^

Potential disadvantages and limitations of this technique include the operator learning curve, anatomic variations in patients, inadequate views of important landmarks, pneumothorax, temporary anesthesia of adjacent nerves (ie, brachial plexus), and intravascular injection of an anesthetic. Prior studies examining nerve blocks of similar nerves found them safe for routine use with no reported complications.^9^^,^^10^ As always, a careful anesthesia plan with a bailout protocol must be in place in case the nerve block is not successful.

## Summary

The SCN and PECS 1 nerve blocks can be used as a primary mode of anesthesia for patients undergoing pre-pectoral CIED implantation to minimize or eliminate the use of intravenous sedation and local anesthetic. Further studies are required to assess the feasibility, safety, and benefits of using this as a routine approach.

## Funding Support and Author Disclosures

The authors have reported that they have no relationships relevant to the contents of this paper to disclose.

## References

[1] Kusumoto F.M., Schoenfeld M.H., Barrett C. (2019). 2018 ACC/AHA/HRS guideline on the evaluation and management of patients with bradycardia and cardiac conduction delay. J Am Coll Cardiol.

[2] Aldridge L.M. (1985). Anaesthetic problems in myotonic dystrophy. Br J Anaesth.

[3] Chen C., Xu J., Huang F. (2013). Conscious sedation and analgesia for cardiac device implantation: anesthesiologist or not?. Int J Cardiol.

[4] Tobias J., Leder M. (2011). Procedural sedation: a review of sedative agents, monitoring, and management of complications. Saudi J Anaesth.

[5] Yang J.K., Char D.S., Motonaga K.S. (2020). Pectoral nerve blocks decrease postoperative pain and opioid use after pacemaker or implantable cardioverter-defibrillator placement in children. Heart Rhythm.

[6] Fujiwara A., Komasawa N., Minami T. (2014). Pectoral nerves (PECS) and intercostal nerve block for cardiac resynchronization therapy device implantation. SpringerPlus.

[7] Bozyel S., Yalnız A., Aksu T., Guler T.E., Genez S. (2019). Ultrasound-guided combined pectoral nerve block and axillary venipuncture for the implantation of cardiac implantable electronic devices. Pacing Clin Electrophysiol.

[8] Alvarez-Bastidas L., Morales-Vera E., Valle-Leal J.G., Marroquín-González J. (2018). Delirium in the elderly patient after anesthesia: associated factors. Colombian Journal of Anesthesiology.

[9] Kesisoglou I., Papavramidis T.S., Michalopoulos N. (2009). Superficial selective cervical plexus block following total thyroidectomy: a randomized trial. Head Neck.

[10] Thomas M., Philip F., Mathew A., Krishna K.J. (2018). Intraoperative pectoral nerve block (Pec) for breast cancer surgery: a randomized controlled trial. J Anaesthesiol Clin Pharmacol.

